# Surgery or Non-surgical Treatment of ≤8 mm Non-small Cell Lung Cancer: A Population-Based Study

**DOI:** 10.3389/fsurg.2021.632561

**Published:** 2021-05-26

**Authors:** Jianfei Shen, Weitao Zhuang, Congcong Xu, Ke Jin, Baofu Chen, Dan Tian, Crispin Hiley, Hiroshi Onishi, Chengchu Zhu, Guibin Qiao

**Affiliations:** ^1^Department of Cardiothoracic Surgery, Taizhou Hospital of Zhejiang Province Affiliated to Wenzhou Medical University, Linhai, China; ^2^Department of Thoracic Surgery, Guangdong Provincial People's Hospital, Guangdong Academy of Medical Sciences, Guangzhou, China; ^3^Shantou University Medical College, Shantou, China; ^4^Cancer Research United Kingdom (CRUK) Lung Cancer Centre of Excellence, University College London, London, United Kingdom; ^5^Department of Radiology, University of Yamanashi, Yamanashi, Japan

**Keywords:** indeterminate pulmonary nodule, non-small cell lung cancer, treatment strategy, sublobar resection, lobectomy

## Abstract

**Background:** Timing for intervention of small indeterminate pulmonary nodules has long been a topic of debate given the low incidence of malignancy and difficulty in obtaining a definite preoperative diagnosis. We sought to determine survival outcomes of surgical and non-surgical managements in non-small cell lung cancer (NSCLC) ≤8 mm, which may provide a reference for prospective decision-making for patients with suspected NSCLC.

**Method:** A total of 1,652 patients with Stage IA NSCLC ≤8 mm were identified from the Surveillance, Epidemiology, and End Results (SEER) database and categorized into surgery and non-surgery groups. Chi-square test, *t*-test and Mann-Whitney U test were used to compare the baseline characteristics between groups. Survival curves were depicted using Kaplan-Meier method and compared by log-rank test. Cox proportional hazard model was used for univariate and multivariate analyses. Adjustment of confounding factors between groups was performed by propensity score matching.

**Results:** The surgery and non-surgery groups included 1,438 and 208 patients, respectively. Patients in surgery group demonstrated superior survival outcome than patients in non-surgery group both before [overall survival (OS): HR, 16.22; 95% CI, 11.48–22.91, *p* < 0.001; cancer-specific survival (CSS): HR, 49.6; 95% CI, 31.09–79.11, *p* < 0.001] and after (OS: HR, 3.12; 95% CI, 2.40–4.05, *p* < 0.001; CSS: HR, 3.85; 95% CI, 2.74–5.40, *p* < 0.001) propensity score matching. The 30-day mortality rates were 3.1 and 12.0% in surgery and non-surgery groups, respectively. Multivariate analysis suggested age, sex, race, tumor size, grade, pathological stage were all independent prognostic factors in patients with ≤8 mm NSCLC. A comparison of surgical resections revealed a survival superiority of lobectomy over sub-lobectomy. In terms of CSS, no statistically significant difference was found between segmentectomy and wedge resection.

**Conclusion:** The current SEER database showed better prognosis of surgical resection than non-surgical treatment in patients with ≤8 mm NSCLC. However, the factors that should be essentially included in the proper propensity-matched analysis, such as comorbidity, cardiopulmonary function and performance status were unavailable and the true superiority or inferiority should be examined further by ongoing randomized trial, especially comparing surgery and stereotactic body irradiation.

## Introduction

With the extensive use of low-dose computed tomography (CT) in lung cancer screening programs, tumors are being detected at smaller sizes and earlier stage than ever before (1). Given that the vast majority of small-sized pulmonary nodules are benign (2), observation with serial CT scans is recommended by updated guidelines and recommendations, with period of follow-up determined by tumor size, density, and patient risks (3, 4). This strategy aims to avoid unnecessary surgery in patients with benign nodules. However, it may cause a dilemma for clinicians by potentially risking delayed diagnosis and treatment in those with malignant nodules, whose growth rate varies widely (5). Rapidly growing cancer may progress from an undetectable to a symptomatic state, or from resectable to unresectable disease between screens (6, 7). The IASLC Lung Cancer Staging Project found a significant difference in 5-year survival between 0.1–1 cm (91%) and 1.1–2 cm (86%) NSCLC, although both groups were pathologically N0M0 and underwent R0 resection (8). This suggests the beneficial outcome of early detection and resection of NSCLC, and watchful waiting may impair the long-term outcome of rapid-growing nodules which are malignant in nature. In this regard, the management plan for small-sized pulmonary nodules with high malignant probability remains controversial.

Differentiation between benign and malignant causes among the incidentally detected pulmonary nodules is certainly the key in clinical practice. Advances in radiological modalities have enabled the estimation of invasiveness and prognostic stratification in early-stage lung cancer (9, 10). The combination of CT findings and a positive PET scan has largely increased the diagnostic accuracy when confirmed with final pathological results (11, 12). Yet, despite its great advantage, PET scan would not be an immediate choice of diagnostic workup given its high cost and concerns about false-positive results in cases of focal pneumonia, granulomas, and tuberculosis, etc. (13, 14). Imaging can never arrive at an exact diagnosis, especially for small-sized NSCLC with diameter of 8 mm or less, in which typical features of malignancy are usually absent (15). In such cases, a certain diagnosis can only be established by tissue sampling. CT-guided percutaneous fine-needle aspiration biopsy (FNAB) has shown a fair diagnostic efficiency for 0.8–1.0 cm nodules (sensitivity, 88%; accuracy, 92%), however, results are inferior for nodules measuring <8 mm (sensitivity, 50%;accuracy, 70%) (16). This procedure is also risky, with 62% of cases complicated by pneumothorax, and 31% requiring thoracostomy tube placement (16). Given this, tissue sampling is not recommended as a diagnostic workup for pulmonary nodules smaller than 8 mm (3), and consequently, they become a gray zone of surgical intervention. It was reported that 27–46% of patients who underwent surgical resection had no preoperative histological diagnosis (17, 18), which could be even higher in small sized pulmonary nodules.

For early-stage NSCLC, previous studies mainly focused on whether sublobar resection would be an oncologically equivalent procedure to lobar resection (19, 20). However, in terms of small indeterminate pulmonary nodules, from a prospective point of view, the question of whether to offer surgical intervention should be addressed before the question of which surgical resection is appropriate can be answered. In this study, we investigated the outcomes of different treatment strategies for NSCLC ≤8 mm in the Surveillance, Epidemiology, and End Results (SEER) database. Hopefully, this retrospective analysis could give us an insight into the management of small-sized NSCLC, and provide survival data for physicians to share with patients when weighing the risks and benefits of surgical and non-surgical therapy for small-sized pulmonary nodules with high malignant probability.

## Patients and Methods

SEER database collects data of cancer patients in 18 areas of the United States and therefore, is highly representative in terms of geography, socioeconomic status, and ethnicity. Clinicopathologic data such as sociodemographic information, features of tumors, and treatment details were retrieved for this retrospective analysis. Informed consent from the study population was not deemed necessary, as the authors had no access to the identities of the patients. The ICD-O-3 histology/behavior code of NSCLC was specified as 8012/3, 8046/3, 8070/3, 8071/3, 8072/3, 8140/3, 8250/3, 8252/3, 8255/3, and 8560/3. We selected all patients with stage IA NSCLC diagnosed between 2000 and 2016, and further identified patients with tumor size ≤8 mm. Patients with two or more primary tumors were only included with their first surgical treatment. Those with inconsistent tumor size and T stage and those with no pathological confirmation of NSCLC were excluded for analysis. All patients were restaged according to the seventh edition of AJCC TNM staging system.

Patients were divided into surgery and non-surgery groups based on their surgical status and were further subclassified for survival analysis according to surgical resections. Among the selected patients, a unique field recording surgical status allowed for identification of reasons for not receiving surgery. Reasons described as surgery “not recommended,” “not recommended, contraindicated due to other conditions,” and “recommended but not performed, patient refused/unknown reason” all fell into the category of “non-surgery.”

Treatment details of the non-surgical group, which may include stereotactic ablative radiotherapy (SABR), radiofrequency ablation (RFA), or simply regular follow-up, were not available from the SEER database. The outcomes of interest in this study were overall survival (OS) and cancer-specific survival (CSS). Median follow-up time was 44 months. Survival outcomes were obtained up until December 31, 2016. OS was defined as the interval from the date of cancer diagnosis to the date of death reported in the registry. CSS was defined as the time from diagnosis to death from lung cancer only. The cutoff time for follow-up was 120 months. Those who survived past December 31, 2016, and those who were alive for longer than 120 months were classified as censored.

## Statistical Analysis

Pearson's chi squared test and Mann-Whiney *U*-test were used for comparison between categorical and ordinal variables, respectively. For continuous variables, age was compared by two-sample *t*-test while tumor grade was compared using Kruskal-Wallis test. Survival was estimated using the Kaplan-Meier method and then compared by log-rank test. The Cox proportional hazard model was used for univariate and multivariate analyses. Multivariate analysis was adjusted for patient age, gender, tumor size, histology, grade, and primary sites. To further adjust for potential confounding factors, a propensity score-matched (1:2, Caliper 0.2) analysis (adjusted variables: age, gender, race, tumor size, histology, grade, and primary sites) was performed to compare OS and CSS in the surgery and non-surgery groups. A *p*-value <0.05 was considered statistically significant, and all statistical tests were two sided. All statistical analyses were carried out using IBM SPSS Statistics 22.0 (IBM Corp., Armonk, NY, USA), and R software version 3.6.3 (R Foundation for Statistical Computing, Vienna, Austria).

## Results

### Clinicopathologic Characteristics

Information of 11,220 patients diagnosed with stage IA NSCLC between 2000 and 2016 were retrieved from SEER database. A total of 1,652 patients with tumor size ≤8 mm who satisfied all the inclusion and exclusion criteria stated above were included for analysis. Baseline clinicopathologic characteristics before and after propensity score matching are reported in Table 1. In the overall cohort, histologic subtypes included squamous cell carcinoma (*N* = 387, 23.4%), adenocarcinoma (*N* = 877, 53.1%), and other subtypes of NSCLC (*N* = 388, 23.5%). The majority of cancers were well-differentiated (*N* = 487, 29.5%) or moderately differentiated (*N* = 527, 31.9%). In addition, the tumors were mainly located in the upper lobe (*N* = 1,064, 64.5%) and the lower lobe (*N* = 445, 26.9%). There were 1,438 patients in the surgery group, with mean tumor size being 6.3 mm, and the rest of the patients who did not undergo with surgery, having a mean tumor size of 5.5 mm. There was no statistical significant difference in gender or race between the surgery and non-surgery groups (Table 1).

**Table 1:** Characteristics of NSCLC patients with tumor size ≤ 8 mm.

	**Whole Cohort^a^**	**Before PSM**			**After PSM**		
**Characteristics**		**Non-surgery**	**Surgery**	***p-*value**	**Non-surgery**	**Surgery**	***p-*value**
	***N* = 1,652, %**	***n* = 208, %**	***n* = 1,438, %**		***n* = 181, %**	***n* = 316, %**	
Age (in years)				<0.001			0.185
Mean ± SD	66.38 ± 9.76	71.65 ± 0.72	65.6 ± 0.25		70.35 ± 10.02	69.18 ± 8.46	
Sex				0.041			0.883
Male	657, 39.8	96, 46.2	557, 38.7		82, 45.3	141, 44.6	
Female	995, 60.2	112, 53.8	881, 61.3		99, 54.7	175, 55.4	
Race				0.676			0.750
White	1,430, 86.6	184, 88.5	1,241, 86.7		162, 89.5	278, 88.0	
Black	123, 7.4	15, 7.2	107, 7.5		12, 6.6	21, 6.6	
Others	99, 6.0	9, 4.3	83, 5.8		7, 3.9	17, 5.4	
Tumor size (mm)				<0.001			0.363
Mean ± SD	6.18 ± 1.98	5.5 ± 0.2	6.3 ± 0.05		5.80 ± 2.11	5.62 ± 2.30	
Histology				0.001			0.594
Squamous cell carcinoma, NOS	387, 23.4	69, 33.2	316, 22.0		62, 34.3	95, 30.1	
Adenocarcinoma, or with mixed subtypes, NOS	877, 53.1	90, 43.3	785, 54.6		77, 42.5	139, 44.0	
Others	388, 23.5	49, 23.6	337, 23.4		42, 23.2	82, 25.9	
Grade				<0.001			0.734
Well-differentiated; Grade I	487, 29.5	25, 26.9	461, 36.8		25,13.8	43, 13.6	
Moderate differentiated; Grade II	527, 31.9	25,26.9	502, 40.0		25, 13.8	59, 18.7	
Poor differentiated; Grade III	316, 19.1	41, 44.1	274, 21.9		41, 22.7	69, 21.8	
Undifferentiated, anaplastic; Grade IV	20, 1.2	2, 2.2	17, 1.4		2, 1.1	3, 0.9	
Unknown	302, 18.3	–	–		88, 48.6	142, 44.9	
Primary site				<0.001			0.652
Upper lobe	1,065, 64.5	123, 59.1	939, 65.3		109, 60.2	195, 61.7	
Middle lobe	100, 6.1	9, 4.3	91, 6.3		8, 4.4	20, 6.3	
Lower lobe	445, 26.9	55, 26.4	388, 27.0		52, 28.7	86, 27.2	
Main bronchus, overlapping lesion and lung, NOS	42, 2.5	21, 10.1	20, 1.4		12, 6.6	15, 4.7	
Survival time (months)				<0.001			<0.001
Mean	105.41	36.8	87.6		37.9	77.8	

a *The surgical status of 6 patients were missing*.

*SD, standard deviation*.

### Survival Outcome and Hospital Mortality

**Figure 1:**
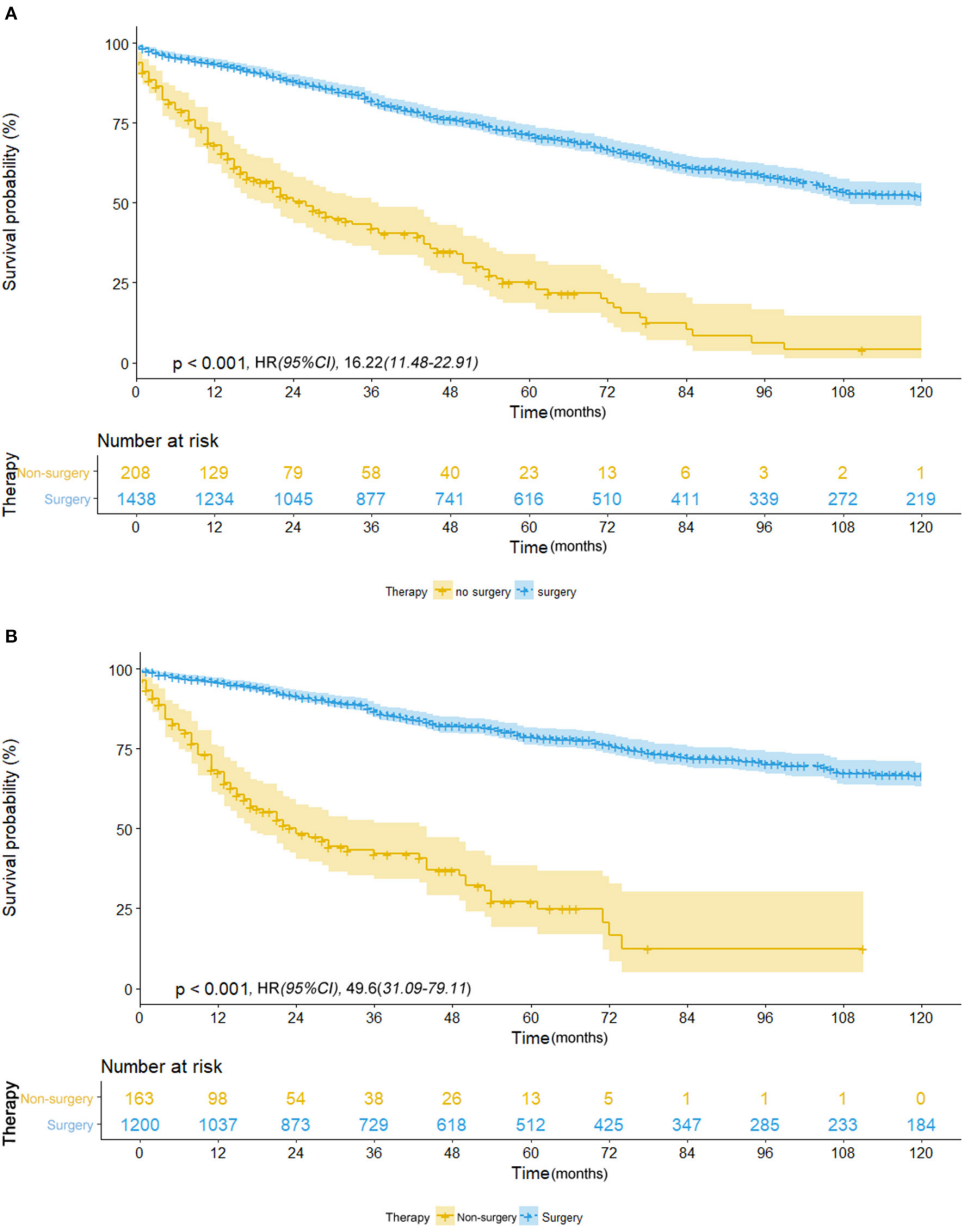
**(A)** Overall survival in patients with NSCLC ≤8 mm who received surgical treatment (blue line) vs. non-surgical treatment (yellow line) before propensity score matching. **(B)** Cancer-specific survival in patients with NSCLC ≤8 mm who received surgical treatment (blue line) vs. non-surgical treatment (yellow line) before propensity score matching.

Of those who received surgery, 835 patients underwent lobectomy and 580 underwent sub-lobectomy (489 wedge resection; 80 segmental resection). The surgical resections of 23 patients were not specified and were therefore not included in survival comparison between the lobectomy and sub-lobectomy groups. Surgery was more likely to be offered for younger patients (*p* < 0.001) and patients with larger tumor size (*p* < 0.001). For patients with ≤8 mm NSCLC, the mean OS and CSS of the surgery group were recorded as 87.6 and 97.0 months, respectively (median OS and CSS were not reached), which was significantly better than those of the non-surgery group (OS: 36.8 months; CSS: 38.6 months) by log-rank test [OS: hazard ratio (HR), 16.22; 95% CI, 11.48–22.91; *p* < 0.001; CSS: HR, 49.6; 95% CI, 31.09–79.11; *p* < 0.001] (Table 2; Figures 1A,B). Survival of 0–1 month calculated from diagnosis was defined as hospital mortality, which served as a parameter for short-term outcome. In the surgery and non-surgery groups, the hospital mortality rate was 3.1% (45/1,438) and 12.0% (25/208), respectively.

**Table 2:** Overall survival and cancer-specific survival of different treatments in patients with tumor size ≤ 8 mm.

	**Before PSM**, ***n*** **=** **1,646**^**a**^						**After PSM**, ***n*** **=** **497**					
**Variables**	**Overall survival**			**Lung cancer-specific survival**			**Overall survival**			**Lung cancer-specific survival (*****n*** **=** **392)**		
	**Mean (months)**	**HR (95% CI)**	***p*-value**	**Mean (months)**	**HR (95% CI)**	***p*-value**	**Median (months)**	**HR (95% CI)**	***p*-value**	**Mean (months)**	**HR (95% CI)**	***p*-value**
Therapy		16.22 (11.48–22.91)	<0.001		49.6 (31.09–79.11)	<0.001		3.12 (2.40–4.05)	<0.001		3.85 (2.74–5.40)	<0.001
Non-surgery	36.8			38.6			27.0			40.5		
Surgery	87.6			97.0			93.0			88.4		
Surgical resection		1.53 (1.26–1.87)	<0.001		1.62 (1.24–2.11)	0.0004						
Sub-lobectomy	81.8			91.8								
Lobectomy	91.9			100.7								
Sub-lobectomy		1.62 (1.08–2.44)	0.021		1.63 (0.97–2.75)	0.064						
Wedge	80.3			90.6								
Segmental	95.1			101.7								

a *The surgical status of 6 patients were missing*.

**Figure 2:**
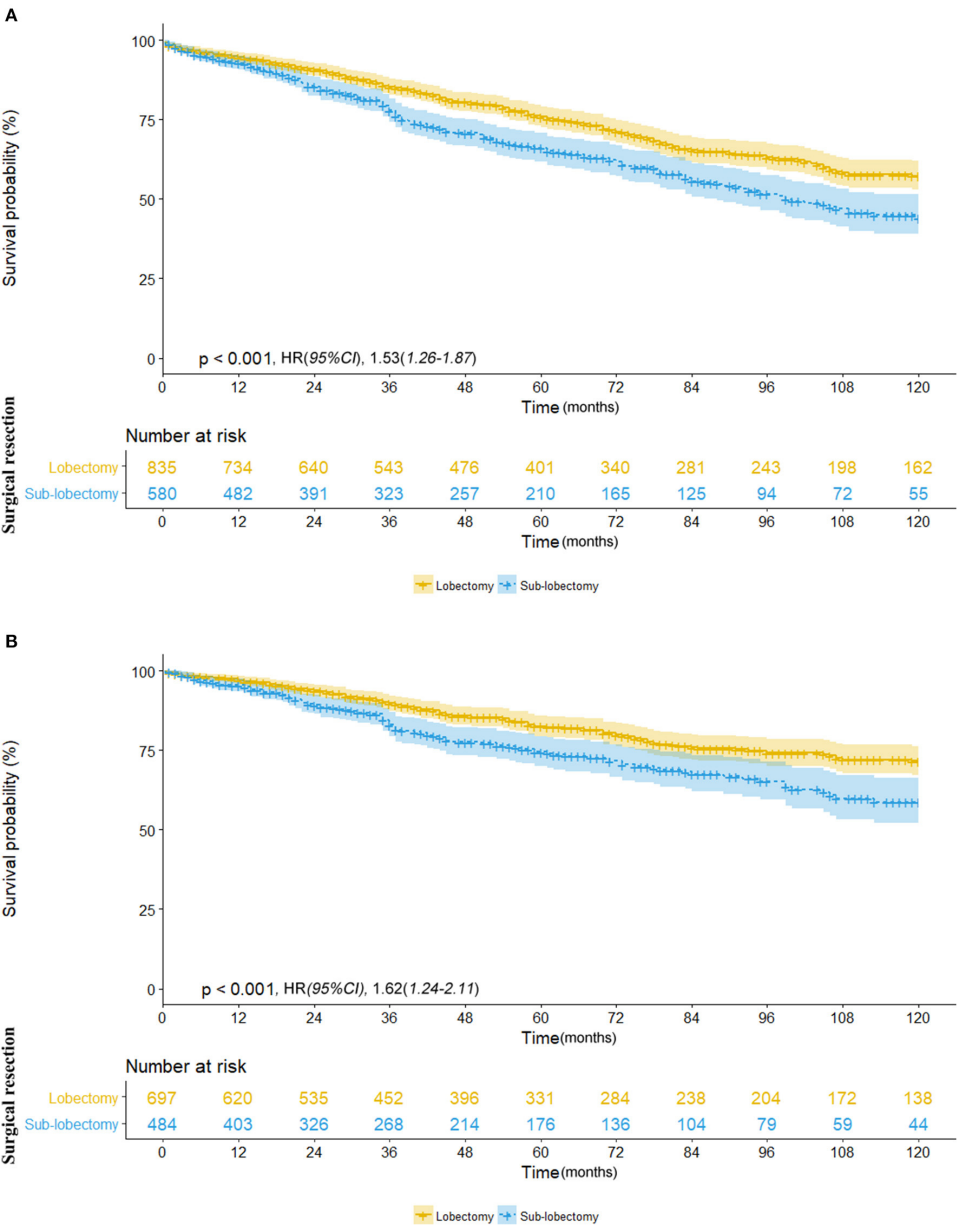
**(A)** Overall survival in patients with NSCLC ≤8 mm who received lobectomy (yellow line) vs. sub-lobectomy (blue line). **(B)** Cancer-specific survival in patients with NSCLC ≤8 mm who received lobectomy (yellow line) vs. sub-lobectomy (blue line).

**Figure 3:**
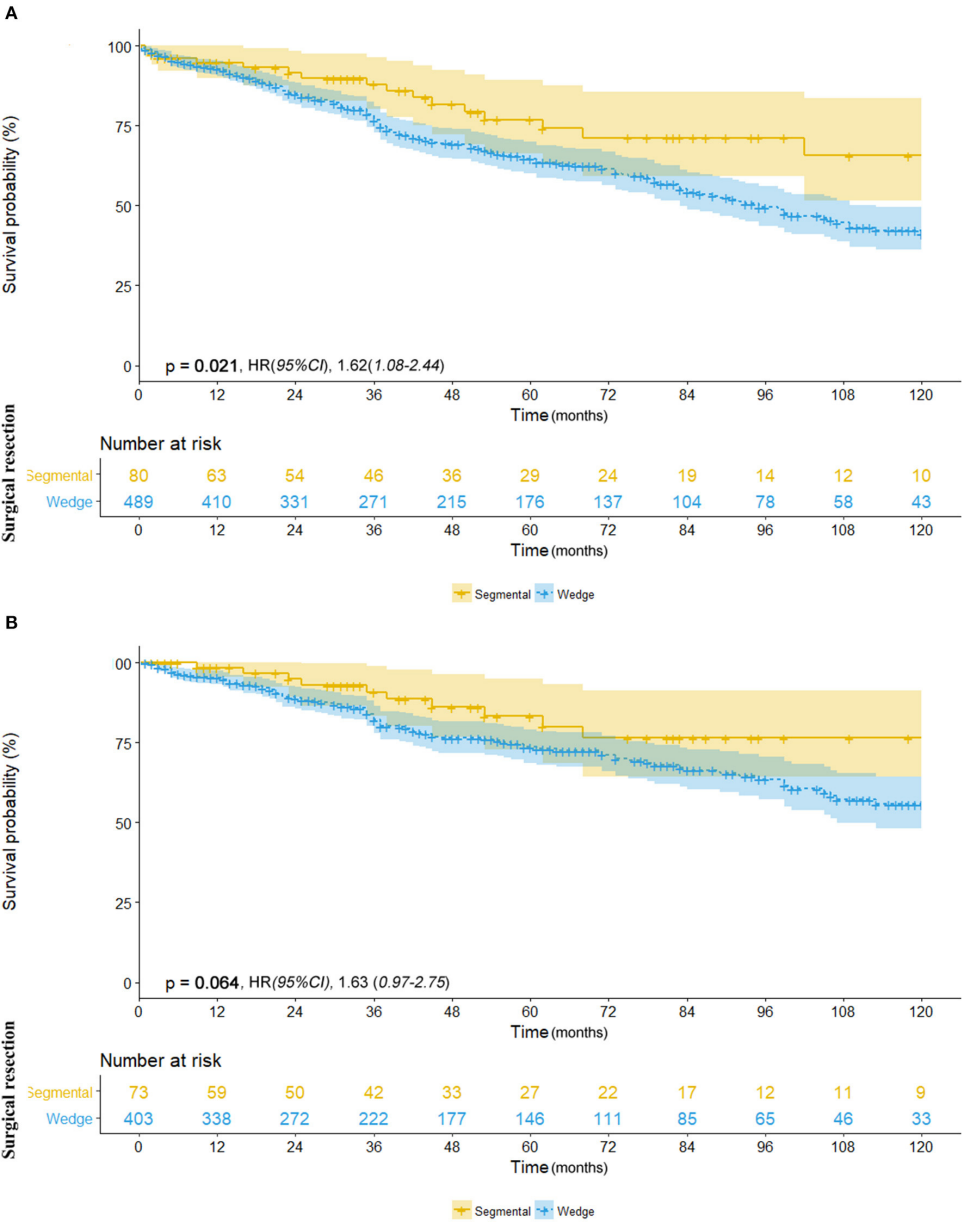
**(A)** Overall survival in patients with NSCLC ≤8 mm who underwent segmentectomy (yellow line) vs. wedge resection (blue line). **(B)** Cancer-specific survival in patients with NSCLC ≤8 mm who underwent segmentectomy (yellow line) vs. wedge resection (blue line).

We further investigated the differences in survival outcomes between lobectomy and sub-lobectomy. Results showed that lobectomy was associated with significant better mean OS (91.9 vs. 81.8 months; HR, 1.53; 95% CI, 1.26–1.87; *p* < 0.001) and CSS (100.7 vs. 91.8 months; HR, 1.62; 95% CI, 1.24–2.11; *p* < 0.001) than sub-lobectomy (Table 2; Figures 2A,B). Further comparison among the sub-lobectomy group yielded better results for segmentectomy vs. wedge resection only in terms of mean OS (95.1 vs. 80.3 months; HR, 1.62; 95% CI, 1.09–2.38; *p* = 0.021) but not CSS (101.7 vs. 90.6 months; HR, 1.63; 95% CI, 0.97–2.75; *p* = 0.064) (Table 2; Figures 3A,B).

### Survival Outcome With Adjustment

**Figure 4:**
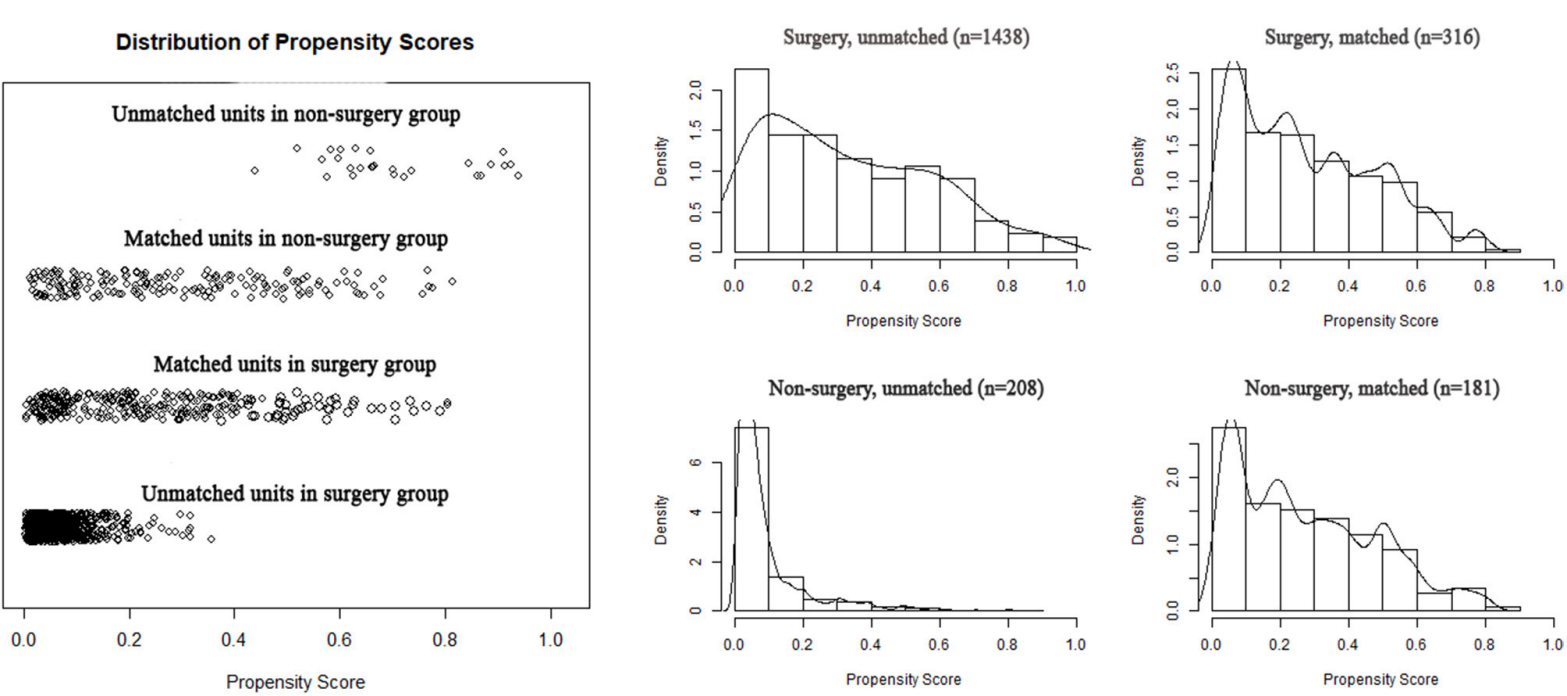
Distribution of propensity score before and after propensity score matching.

**Figure 5:**
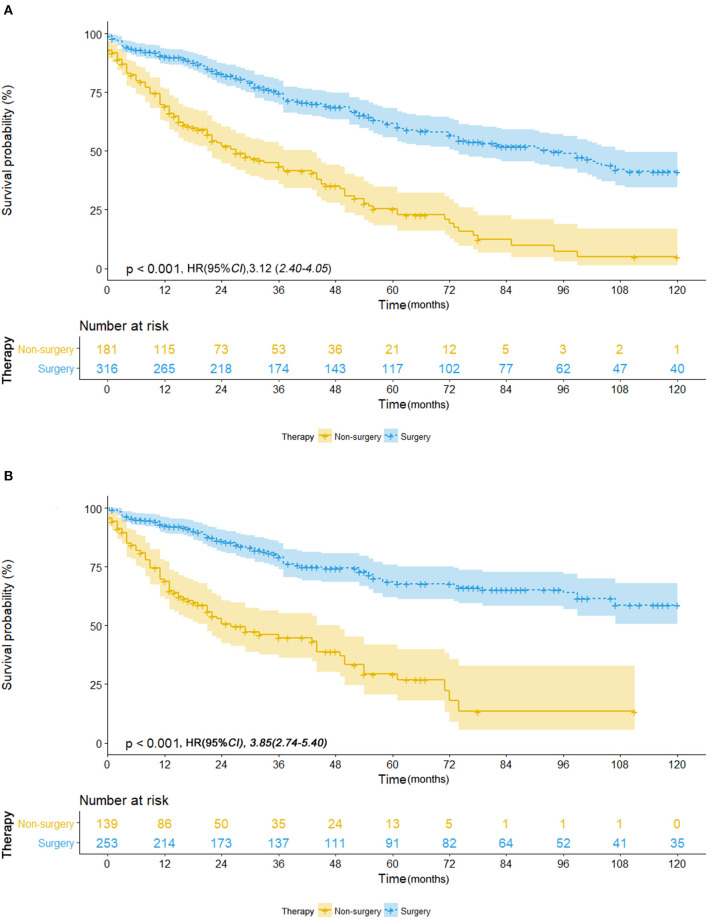
**(A)** Overall survival in patients with NSCLC ≤ 8 mm who received surgical treatment (blue line) vs. non-surgical treatment (yellow line) after propensity score matching. **(B)** Cancer-specific survival in patients with NSCLC ≤ 8 mm who received surgical treatment (blue line) vs. non-surgical treatment (yellow line) after propensity score matching.

To reduce the potential bias due to an imbalance between the surgery and non-surgery groups regarding patient age, gender, race, tumor size, histology, grade, and primary sites, propensity score matching was performed. This procedure resulted in the exclusion of 1,149 patients (1,122 patients in the surgery group and 27 in the non-surgery group). Figure 4 depicts the distribution of the propensity scores of the two patient groups before and after the matching procedure. An imbalance in patient age, tumor grade, and primary sites before matching was adjusted (Table 1). We then sought to compare survival between the matched groups, which still revealed an advantage of both median OS (93.0 vs. 27.0 months; HR, 3.12; 95% CI, 2.40–4.05; *p* < 0.001) and mean CSS (88.4 vs. 40.5 months; HR, 3.85; 95% CI, 2.74–5.40; *p* < 0.001) in the surgery group (Table 2; Figures 5A,B).

**Figure 6:**
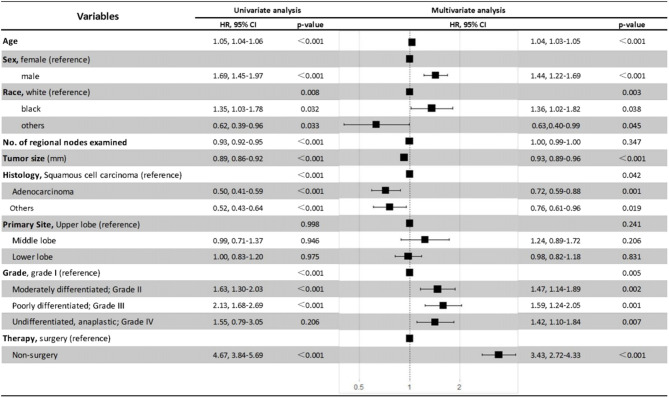
Univariate and multivariate analysis for overall survival of the patients.

The Cox proportional hazards regression model was also used to minimize the interference of potential confounding factors (Figure 6). With the adjustment of necessary patient and tumor variables, non-surgery was found to be independently associated with poorer OS and CSS than surgery in patients with NSCLC ≤8 mm in multivariate analysis (HR, 3.43; 95% CI, 2.72–4.33; *P* < 0.001). Moreover, the results also demonstrated that being older, male, or black, or having larger tumor size, squamous cell carcinoma, or advanced tumor grade were all independent negative prognostic factors for OS in patients with ≤8 mm NSCLC (Figure 6).

## Discussion

Tumor size has long been recognized as a primary descriptor and strong prognostic factor for lung cancer (21). For small-sized, early-stage NSCLC, surgical resection remains the standard of care; however, its role is now being challenged by emerging alternative measures such as SABR, especially for patients with advanced age and high surgical risks (22, 23). Here, we sought to determine the outcomes of surgical and non-surgical treatments of ≤ 8 mm NSCLC, which is a gray zone of management before a histologically confirmed diagnosis can be obtained. Patient diagnosis in our study was mostly confirmed by positive histology or positive exfoliative cytology. But still, more than 10% of patients did not receive the standard care. Among this group, 159/207 patients who were not recommended for surgery did not have any clearly known contraindications. Previous studies also reported that at least 15–20% of stage I NSCLC patients were unable to undergo or refuse definitive surgical resection (24, 25). Common reasons for patients not receiving surgery are insufficient pulmonary reserve and medical comorbidities (26). From a prospective standpoint, it is not surprising that many patients with small indeterminate high-risk nodules are not recommended for or directly refuse surgery when an exact diagnosis cannot be established.

There is limited evidence for the management of small-sized, high malignancy probability pulmonary nodules. In view of the low incidence of malignant nodules in CT screening (2, 27), some physicians proposed to lengthen the interval between CT surveillance and to increase the tumor size threshold for lung cancer diagnosis, in order to reduce over-diagnosis and unnecessary surgery (27). However, as clinicians need to make decisions prospectively for each individual patient, the benefits of early detection and treatment and the potential risks of unnecessary surgery should be carefully balanced. The best way to answer this question would be a prospective randomized trial to compare the clinical outcomes of different treatment strategies for small-sized, indeterminate nodules. However, so far no high-quality study has addressed this issue. In this study, we investigated the related evidence from an unusual perspective, from where we can come up with suggestions for treatment of ≤8 mm NSCLC, as well as provide a reference for suspected NSCLC without pathological confirmation.

In the current study, it has been well-demonstrated that survival outcome of surgery was significantly favorable compared with that of non-surgical managements when the nodules were malignant in nature. We arrived at the same conclusion after propensity score matching, which helped to adjust many potential confounders and thus achieved more reliability when conclusions were drawn. This finding is in line with previous retrospective studies which assessed the role of multimodality therapy for operable NSCLC (23, 28). Shirvani reported outcomes from the SEER database of 9,093 stage I NSCLC patients, revealing an increased 90-day mortality (4.0% vs. 1.3%, *p* = 0.08) but a decreased 3-year mortality rate (25% vs. 45.1%, *p* < 0.001) with lobectomy when compared with SABR (23). Puri reported outcomes of 117,618 stage I NSCLC patients on the National Cancer Database (NCDB) and showed a survival advantage for surgical resection compared with SABR both before (mOS: 68.4 vs. 33.3 months, *p* < 0.001) and after (mOS: 62.3 vs. 33.1 months, *p* < 0.001) propensity score matching (26). RFA, which has mainly been studied in medically inoperable NSCLC, achieved survival outcomes comparable with those of SABR in similar patients (29, 30). In terms of short-term outcome, the 30-day mortality rate of surgery in our study is similar to that of a National Cancer Data Base study assessing 124,418 major lung resections (3.1% and 2.8%, respectively) (31). However, our study revealed a significantly higher 30-day mortality rate in the non-surgical group compared with the aforementioned studies (23, 28, 30), which may be due to the poorer performance status or possible comorbidities of the non-surgical patients in our study.

Lobectomy has been a standard surgical procedure for early-stage NSCLC since 1995 (32). With increasing detection of small-sized NSCLC and the significantly different 5-year survival rates observed in ≤1 cm and 1–2 cm NSCLC (91% vs. 86% when pathologically staged, and 92% vs. 83% when clinically staged) (8), the eighth edition of the TNM classification for lung cancer further subclassified the former T1a disease into two subgroups at the cutpoint of 1 cm (33). Upon this change, Dai et al. performed a population-based study and found an OS and CSS superiority of lobectomy over sub-lobectomy in both ≤1 cm and 1–2 cm subgroups (20). In particular, wedge resection achieved an oncologically equivalent outcome to segmentectomy in ≤1 cm NSCLC (20). This is consistent with the results found in our study, where ≤8 mm NSCLC can be regarded as a sub-population of NSCLC with size ≤1 cm. However, another SEER-based study using propensity score matching found no significant difference between lobectomy and limited resection in subcentimeter NSCLC (OS: HR 1.12; 95% CI, 0.93–1.35; CSS: HR, 1.24; 95% CI, 0.95–1.61) (19). Different conclusions between studies based on the same database can be contributed by the varied sample sizes, different statistical methodologies, and the selection bias intrinsically accompanying retrospective studies. To draw a more persuasive conclusion, randomized controlled trials have been conducted to compare lobectomy with limited resection in the United States (CALBG 140503) and in Japan (JCOG0802/WJOG4607L), and preliminary results about perioperative safety outcomes were recently released (34, 35). However, survival data are not yet mature in both studies. CALGB/Alliance 140503 study reported that an adverse event of any grade occurred in 54% and 51% of patients who underwent lobectomy and sub-lobectomy, respectively (34). Perioperative safety results from the JCOG 0802 revealed zero treatment-related mortality at either 30 or 90 days among 552 segmentectomy and 554 lobectomy, with grade 2 or greater adverse events occurring in 27.4% and 26.2% of patients, respectively (35). These data may address the concerns about impaired perioperative safety to adopt an extended resection. With all these studies that have investigated the short-term and long-term outcomes, a best treatment strategy can finally be established for early-stage NSCLC.

Small-sized NSCLC is not automatically equal to an early-stage disease. Hattori reported 10.6% of nodal involvement and 15.1% of recurrence in the subcentimeter pure-solid NSCLC (9), which indicates that radiological solid lesions with a size of ≤1 cm should be positively treated, regardless of their small size. Goldwasser reported that 32.1% of male and 24.2% of female NSCLC patients were found to have distant metastatic disease with diameter of primary site ≤15 mm (36). He then established a mathematical model to estimate the tumor size at cure threshold based on the SEER database, and a size of 5–15 mm was recommended for intervention before progression to an incurable disease (36). From a prospective standpoint, we suggest that both indication and eligibility for surgery should be actively evaluated for nodules ≤8 mm, especially for those with a high pretest probability of malignancy. The characteristics to define suspected NSCLC include older age, family history of lung cancer, smoking history, emphysema, larger nodular size, location in the upper lobe, part-solid density, and speculation (2). Assessment of likelihood of malignancy can also be facilitated by PET/CT, CT-guided percutaneous biopsy, and even new techniques such as radiomics nomogram or 3D deep learning (10, 37, 38).

The findings in our study might infer an acceptable benefit over risk of surgical resection for pulmonary nodules ≤8 mm with a high protest probability of malignancy. According to guideline from The Fleischner Society, CT surveillance is recommended for at least 6 months later and then for another 18–24 months for solid nodules ≤8 mm (3). However, it was reported that only 29% and 48% of patients completed at least one recommended follow-up scan (39, 40), and only 58.8% of radiologists employed the rules of the guidelines in clinical practice (41). This casual adherence to surveillance regimen may impair the benefits that stem from early diagnosis and treatment of lung cancer. Given this, physicians need to enhance their vigilance in decision-making when coping with highly suspected malignant nodules.

Our study has some strengths and limitations when compared to previous publications. We are the first study to include NSCLC with size ≤8 mm, the definite diagnosis of which is difficult to obtain pre-operatively and therefore, to a degree, its findings can be generalized to small-sized, high malignancy probability nodules. SEER is a nationwide database that reflects different practice environments in the real world and thus has good generality of the population. Another advantage of our study is the large sample size compared with institutional studies which may be underpowered due to insufficient sample for analysis. The primary limitation of our study is the possible selection bias in treatment allocation brought by its retrospective nature. To attempt to minimize this effect, propensity score matching was used to preclude possible confounders. Despite balanced clinical factors in the propensity-matched groups, our study may still be subject to undocumented differences such as comorbidities, performance status, cardiopulmonary function, ratio of ground glass opacity, etc. Moreover, patients in non-surgical group may be underestimated in clinical staging, which contributed to a worse survival outcome than patients undergoing systemic lymphadenectomy. In these circumstances, the unfavorable effect of non-surgical managements may be overstated. Furthermore, the SEER database does not specify non-surgical treatments, resulting in significant intragroup heterogeneity.

## Conclusion

Surgical intervention was associated with significantly better survival than non-surgical management in patients with ≤8 mm NSCLC. Lobectomy demonstrated a survival superiority over sub-lobectomy, and thus it remains the standard of care for patients who can tolerate the procedure. In terms of cancer-specific survival, decision between segmentectomy and wedge resection is not necessary for NSCLC ≤8 mm, and it can be determined by the location of tumors, experience of surgeons, and personal preference of patients. From a prospective viewpoint, indication and eligibility for surgery should be carefully evaluated for small-sized indeterminate nodules with high pretest probability of malignancy. Once fulfilled, it should be treated in the same way as malignancy and follow the recommendation stated above, in order to prevent early-stage disease progression and pursue a better survival outcome.

## Data Availability Statement

The original data presented in the study can be freely downloaded from the Surveillance, Epidemiology, and End Results (SEER) database, further inquiries can be directed to the corresponding author/s.

## Ethics Statement

Written informed consent for participation was not required for this study in accordance with the national legislation and the institutional requirements.

## Author Contributions

JS, WZ, and CX: conceptualization, methodology, and data acquisition. CZ and GQ: administrative support and supervision. All authors contributed to the statistical analysis, manuscript drafting, editing, and final approval for publication.

## Conflict of Interest

The authors declare that the research was conducted in the absence of any commercial or financial relationships that could be construed as a potential conflict of interest.
